# Hereditary Protein S Deficiency With an Extensive Femoral Artery Thrombosis

**DOI:** 10.7759/cureus.51355

**Published:** 2023-12-30

**Authors:** Noor Haslina Mohd Noor, Nurul Anis Che Anuar, Nur Ilyia Syazwani Saidin, Salfarina Iberahim, Abdul Hanan Abdullah

**Affiliations:** 1 Department of Hematology, School of Medical Sciences, Universiti Sains Malaysia, Kota Bharu, MYS; 2 Department of Internal Medicine, School of Medical Sciences, Universiti Sains Malaysia, Kota Bharu, MYS

**Keywords:** haemostasis, hereditary thrombophilia, hereditary protein s deficiency, blood coagulation disorder, atrial thrombosis

## Abstract

Protein S (PS) deficiency is widely recognized for its connection to venous thromboembolism risk. However, the relation between PS deficiency and arterial thrombotic events (ATEs) remains uncertain. Here, we report a patient who experienced an ATE with a family history of PS deficiency. We highlight an attention to the issues related to the management of arterial thrombotic events and discuss the potential use of antiplatelet therapy as a treatment option for a specific group of patients diagnosed with PS deficiency.

## Introduction

Discovered in 1979 within Seattle, Washington, protein S (PS) acquired its name from the city of its discovery. One of its primary roles involves assisting activated protein C (APC) in the regulation of activated factor V (FVa) and activated factor VII (FVIIa). When there is a deficiency in PS, it leads to an inability to effectively manage coagulation, resulting in excessive blood clot formation (thrombophilia) and the occurrence of venous thromboembolism (VTE) [1].

PS is a plasma glycoprotein acknowledged for its inherent anticoagulant properties. Its function as a cofactor for APC enhances the proteolytic cleavage of FVa and FVIIIa, thereby reducing thrombin generation. Moreover, PS acts as a cofactor for tissue factor pathway inhibitor (TFPI), leading to the inactivation of FVIIa [2]. Recent studies have emphasized PS’s ability to deactivate FIXa as well [3].

Around 60-70% of plasma PS is bound to complement factor 4b, leaving the remainder unbound, termed as free PS. The active anticoagulant activity resides within the free PS, whereas bound PS exhibits limited anticoagulation. Consequently, screening for PS deficiency typically involves assessing free PS antigen levels or its functional activity [4].

PS deficiency commonly manifests as VTE and recurrent pregnancy loss, with arterial thrombosis being a rare occurrence [1]. Here, we present a case involving inherited PS deficiency resulting in extensive arterial thrombosis of the bilateral lower limb.

## Case presentation

A 29-year-old male, an active cigarette smoker, smoking one pack per day for the past 10 years, presented with right toes blackish discoloration, right calf throbbing pain, and numbness for one week. The calf pain was intensified with walking but eased by rest. There was no fever or preceding trauma. He experienced myocardial infarction three months prior to this presentation. He underwent angiogram and was treated accordingly. No thrombophilia work up done at this time. No history of VTE in the family was stated. Apart from myocardial infarction, he has no other chronic medical illness, such as diabetes mellitus or hypertension.

Upon examination, the patient’s vital signs were normal. Assessment of lower limb revealed pallor, coldness, and reduced sensation on the right side. The right popliteal artery, dorsalis pedis artery, and posterior tibialis artery were not palpable and not detectable by the Doppler ultrasound scan. These findings raised suspicion of acute limb ischemia.

**Figure 1:**
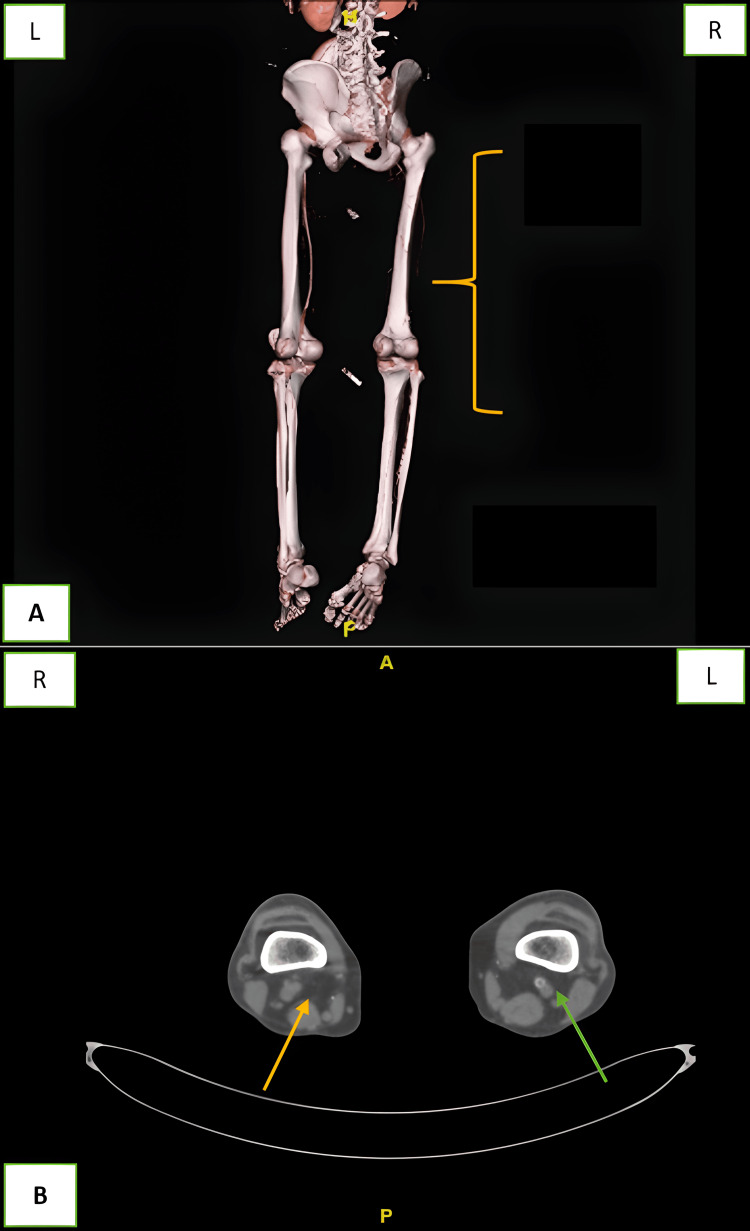
CT angiography of lower limbs. (A) 3D view showing that the right superficial femoral artery (SFA) is not opacified till the popliteal artery (yellow mark) due to thrombosis. (B) Axial view of the proximal thigh showing that the right SFA is not opacified (arrow yellow) but the left SFA is opacified (arrow green). R: right, L: left, A: anterior, P: posterior.

**Figure 2:**
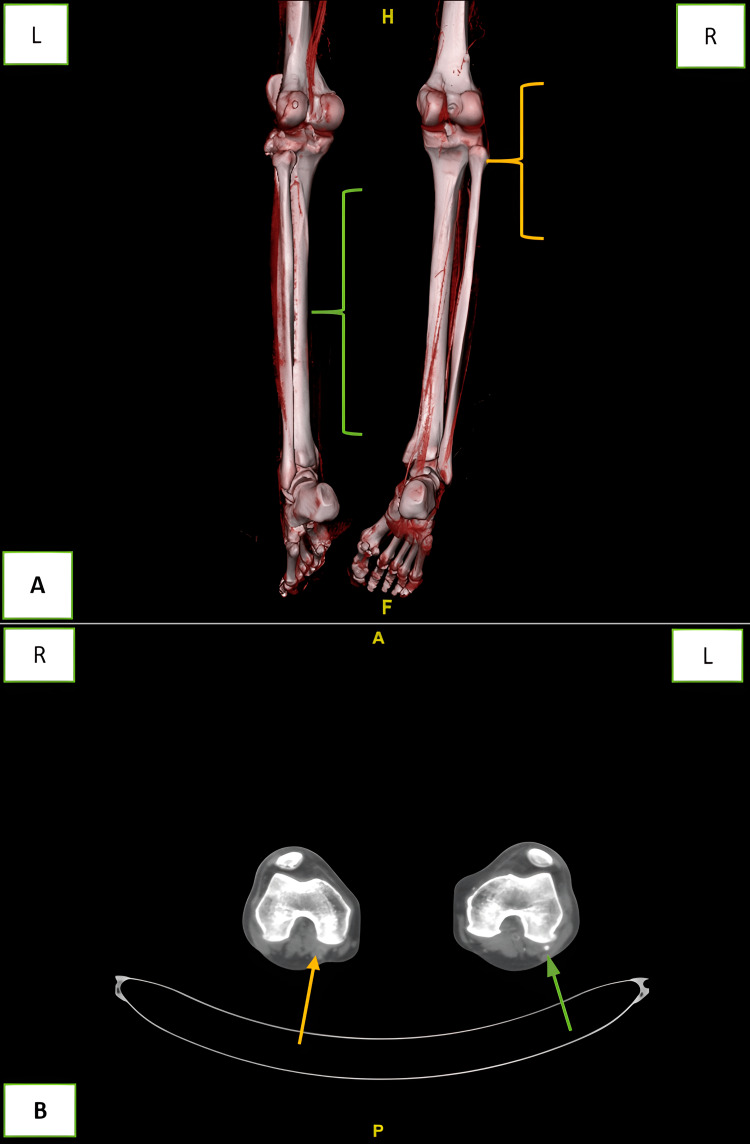
(A) 3D view of the ankle to knee showing a complete thrombosis of the right popliteal artery (yellow mark). The left tibioperoneal trunk and anterior tibial artery are opacified but small in caliber (green mark). (B) Axial view of the distal femur showing complete thrombosis of the right popliteal artery as opposed to the opacification of the left popliteal artery (arrow green). R: right, L: left, A: anterior, P: posterior.

Immediate computed tomography angiography (CTA) of the lower limb was done and revealed complete thrombosis of right superficial femoral and popliteal artery (Figures 1, 2). An attempted interventional angioplasty for the right side was unsuccessful due to the extensive occlusion. Subsequently, the right lower limb thrombectomy and angioplasty were performed, but the procedure failed to establish the distal flow of the right superficial femoral artery. Following unsuccessful radiological intervention, the patient underwent a right below-knee amputation .

In view of severe vascular occlusion, an urgent thrombophilia study was sent and revealed a low free PS antigen level. The total PS antigen and PS activity was not tested due to unavailability of the reagent. Protein C, anti-thrombin activity, and activated protein C resistance (APCR)-Factor V Leiden were normal. The lupus anticoagulant (DRVVT) and anticardiolipin were not detected, thus excluding the antiphospholipid syndromes (Table 1). Anti-beta-2-glycoprotein I antibodies was not done due to unavailability of the test at our hospital. Other laboratory tests, including full blood count, coagulation, renal, and liver profiles, were normal.

**Table 1:** Thrombophilia workup of the patient.

Test	Result	Reference range
Protein C activity	148%	70-140
Free protein S antigen	9.2%	72.2-123.3
Antithrombin activity	155%	83-128
APCR-V Leiden	2.44	Ratio>2.1
Lupus anticoagulant and anti-cardiolipin	Not detected	

**Figure 3:**
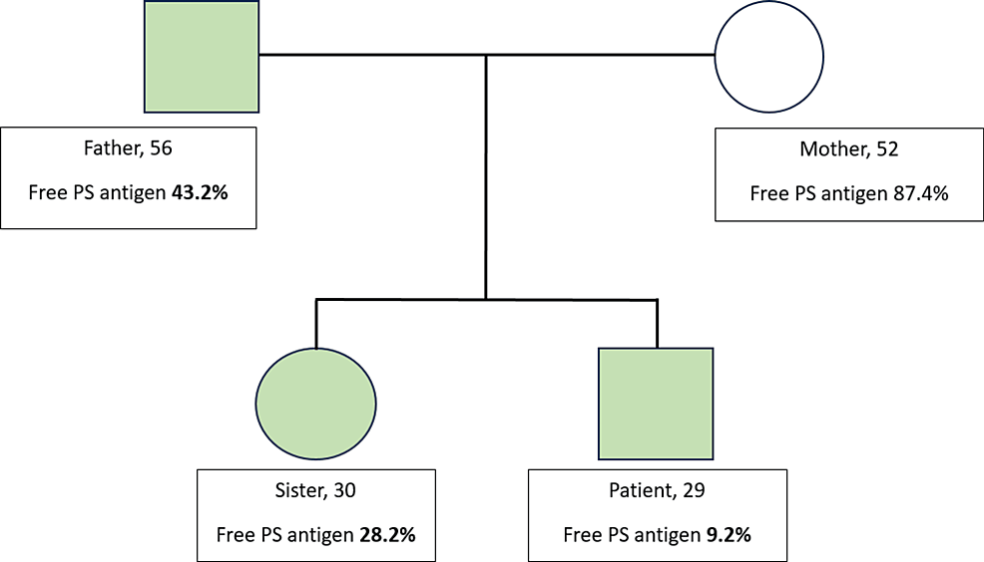
Family pedigree for free PS antigen level screening.

Suspecting inherited PS deficiency, family screening was done and found a low protein S level in the father and sister (Figure 3). However, none of them exhibited any symptoms. They were not put on any prophylaxis treatment. With this additional information, he was then diagnosed with hereditary PS deficiency, most probably type 1 or type 3. This patient did not undergo genetic testing, but such a testing could be beneficial for establishing a conclusive diagnosis. He was started with double antiplatelet therapy (tablet clopidogrel 75 mg, tablet cardiprin 100 mg) and subcutaneous fondaparinux 2.5 mg daily for thromboembolism prophylaxis. He was recommended for counseling following a below-knee amputation and advised to undergo regular physiotherapy after being discharged home. During the follow-up, he remained well with no recurrent thrombosis.

## Discussion

PS deficiency is an uncommon condition characterized by reduced PS activity, a serine protease present in blood plasma that holds intricate roles in coagulation, inflammation, and apoptosis (VTE) [1]. There are three distinct forms of PS deficiency. Type 1 presents as a quantitative defect with low levels of total PS and free PS, along with diminished PS activity. Type 2 involves decreased PS activity, while total PS and free PS antigens remain normal. Type 3 exhibits a quantitative defect with normal total PS levels but reduced free PS levels and PS activity [1].

The age at which thrombosis initiates differs between heterozygous or homozygous states. Most instances of venous thrombosis heterozygous PS deficiency arise in individuals under the age of 40-45 years. PS deficiency is five to 10 times more prevalent in Japanese populations than in Caucasians [5]. In almost 90% of cases, venous thrombosis is the predominant presentation, typically showcasing as unilateral leg or calf swelling accompanied by mild or moderate pain [1]. While PS deficiency is associated with a well-documented risk of VTE, its direct link to arterial thrombotic events (ATEs) remains unclear. It may be that individuals are more likely to have a thrombotic event during a dip in protein S levels, but the exact pathophysiology in this regard remains controversial [5,6].

According to Bakhtawar et al., individuals with PS deficiency (but not antithrombin deficiency) exhibit a higher ATE risk before the age of 55 [6]. Nearly 300 different mutations have been identified in the PS gene (PROS1), spanning all exons, the promoter region, and introns [3]. Most of these alterations are missense mutations, although various other mutation types have been documented. Hereditary PS deficiency stems from mutations in the PROS1 gene, an autosomal disorder. Approximately 50% of cases are identified through conventional polymerase chain reaction (PCR)-based mutation detection [7]. In a study by Zhao et al., sequencing analysis revealed a mutation (c.1486_1490delGATTA) on exon 12 of the PROS1 gene, a frameshift mutation converting Asp496 in the precursor PS into a termination codon, associated with low PS activity [8]. Andersen et al. reported heterozygosity for four novel missense mutations (W108C, W342R, E349K, and L485S) and one novel 4 bp deletion affecting codons 632-633 in the PROS1 gene within Danish families with PS type I or III deficiency [9].

The management of acute thrombosis in individuals with heritable PS deficiency aligns with that of acute VTE in general. Anticoagulant therapies, such as heparin, whether low-molecular-weight heparin (LMWH) or unfractionated, vitamin K antagonist (VKA), or direct oral anticoagulant (DOAC) administration, are utilized. In acute events, the initial treatment involves heparin, whether unfractionated heparin or LMWH, followed by VKA or DOAC. While VKA was previously the preferred drug, DOACs have gained preference due to their safety profile and efficacy [1]. Lifelong therapy is recommended for patients with heritable PS deficiency who experience recurrent or life-threatening thrombotic events. Considering a history of myocardial infarction three months prior, potentially linked to heritable PS deficiency, lifelong anticoagulants and the use of antiplatelet therapy as prophylaxis may prove beneficial in this patient [10].

## Conclusions

PS deficiency is one of a group of inherited disorders termed hereditary thrombotic disease, which may have serious implications for patient morbidity and mortality. PS deficiency is among the factors contributing to thrombotic disorders that warrant attention, particularly in young individuals with a background of DVT or other vascular thromboses. Isolated PS deficiency can lead to catastrophic thrombosis, if not recognized early and managed effectively. This case highlights the significance of including PS deficiency as a potential consideration when assessing patients with arterial thrombosis.
